# Impaired mitophagy in Fanconi anemia is dependent on mitochondrial fission

**DOI:** 10.18632/oncotarget.11161

**Published:** 2016-08-09

**Authors:** Pavithra Shyamsunder, Milan Esner, Maunish Barvalia, Yu Jun Wu, Tomáš Loja, Huat Bay Boon, Matilde E. Lleonart, Rama S Verma, Lumir Krejci, Alex Lyakhovich

**Affiliations:** ^1^ Cancer Science Institute of Singapore, National University of Singapore, Singapore; ^2^ Stem Cell and Molecular Biology Laboratory, Department of Biotechnology, Indian Institute of Technology Madras, Chennai, India; ^3^ Department of Histology and Embryology, Faculty of Medicine, Masaryk University, Brno, Czech Republic; ^4^ Indian Institute of Technology Madras, Chennai, India; ^5^ Yong Loo Lin School of Medicine, Department of Anatomy, National University of Singapore, Singapore; ^6^ Central European Institute of Technology, Masaryk University, Brno, Czech Republic; ^7^ Translational Research in Cancer Stem Cells, Vall d'Hebron Institut de Recerca (VHIR), Barcelona, Spain; ^8^ Department of Biology, Faculty of Medicine, Masaryk University, Brno, Czech Republic; ^9^ ICRC- FNUSA, International Clinical Research Center and St. Anne's University Hospital Brno, Brno, Czech Republic; ^10^ Current Address: Department of Microbiology and Immunology, University of British Columbia, Life Sciences Institute, Vancouver, British Columbia, Canada

**Keywords:** mitophagy, impaired autophagy, Fanconi anemia, ROS, oxidative stress

## Abstract

Fanconi anemia (FA) is a rare genetic disorder associated with bone-marrow failure, genome instability and cancer predisposition. Recently, we and others have demonstrated dysfunctional mitochondria with morphological alterations in FA cells accompanied by high reactive oxygen species (ROS) levels. Mitochondrial morphology is regulated by continuous fusion and fission events and the misbalance between these two is often accompanied by autophagy. Here, we provide evidence of impaired autophagy in FA. We demonstrate that FA cells have increased number of autophagic (presumably mitophagic) events and accumulate dysfunctional mitochondria due to an impaired ability to degrade them. Moreover, mitochondrial fission accompanied by oxidative stress (OS) is a prerequisite condition for mitophagy in FA and blocking this pathway may release autophagic machinery to clear dysfunctional mitochondria.

## INTRODUCTION

FA is a rare genetic disorder associated with bone-marrow failure, genome instability and cancer predisposition [1]. The current state of knowledge on FA pathway relies on at least 16 genes corresponding to the FA genetic subgroups FA-A –Q and several FA-like genes [2]. Biallelic mutations in any one of these genes except for the X-linked FANCB, leads to FA. Another line of studies, dating back to 1980's, has provided consistent evidence for a role of oxidative stress (OS) in FA phenotype, such as excess oxygen sensitivity [3–5], accumulation of oxidative DNA damage and other anomalies of RedOx endpoints [6, 7]. Most notably, direct implications of FA proteins in RedOx pathways have also been reported [8]. Recent observations from our group and others of morphological changes in FA mitochondria accompanied by significant reduction in ATP synthesis, low mitochondrial membrane potential (ΔΨm), and decreased respiration capacity, suggest mitochondrial dysfunction (MDF) in FA [9].

Mitochondrial morphology is regulated by continuous fusion and fission events, two opposing, but coordinated processes that determine mitochondrial content, structure, and are essential for maintaining normal mitochondrial function [10, 11]. The misbalance between mitochondrial fission and fusion is often accompanied by autophagy, a highly regulated process wherein cells remove dysfunctional organelles via specialized structures called autophagosomes [12]. Mitophagy, a selective form of autophagy, which clears the cells of dysfunctional mitochondria, may link accumulated ROS, MDF and pro-inflammatory status in FA [13]. Recent data indicate that when the capacity to undergo mitochondrial biogenesis, is dysregulated, it plays a key role in determining cell survival following mitophagy-inducing injuries and may predispose cells to cancer [14].

Here, we provide evidence of impaired mitophagy in FA, a phenomenon linked to increased susceptibility to proapoptotic insults [15]. We demonstrate that FA cells have increased number of autophagic (presumably mitophagic) events and retain dysfunctional mitochondria due to an impaired ability to degrade them. Moreover, mitochondrial fission accompanied by OS is a prerequisite condition for mitophagy in FA and blocking this pathway may release autophagic machinery to clear dysfunctional mitochondria. We speculate that defective mitophagy, especially at an early stage of embryonic development may explain FA cancer predisposition at later stages.

## RESULTS

### Mitochondrial fission is accompanied by autophagy in FA cells

**Figure 1:**
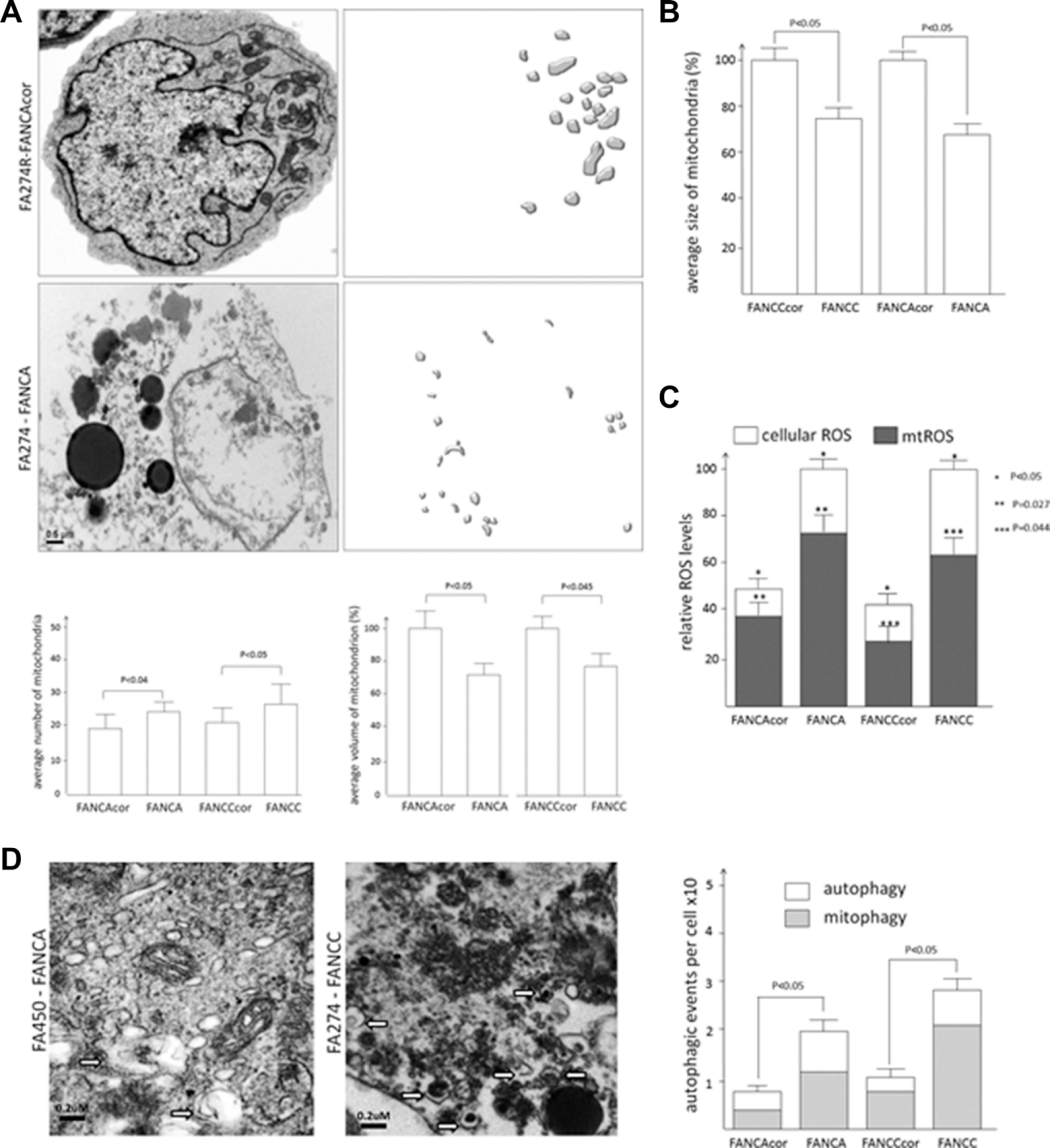
Mitochondrial volume, number and autophagic events in FA and FA corrected cells (**A**) Representative TEM image of FANCA deficient and proficient cells. The average number of mitochondria per image per cell and average mitochondrial volume per cell were calculated upon 3D reconstruction of mitochondrial shapes (right panel). TEM was performed essentially as described previously (9). TEM images from ~9 serial sections 0.2 μm thick were stacked, aligned, and 3D reconstructed. Eight random sections were taken into analysis for each cell lines. Data represent average ± SEM. The full description of cell lines and culturing conditions is provided in Supplementary Table S1B. (**B**) FACS measurement of mitochondrial size from corresponding FA samples. Mitochondrial fractionation has been performed essentially as described earlier (9) and the average size of mitochondria was estimated by comparison with the set of bids with predefined sizes. FA mitochondria were compared as percentage difference, where FAcor mitochondria represented 100%; (**C**) Total cellular and mitochondrial ROS (mtROS) levels. Measurements of intracellular ROS were performed with fluorogenic dye H2DCF-DA and mitochondrial ROS was detected with MitoSOX red reagent following procedure described previously (9). (**D**) Representative TEM images of FA cells with ×60000 resolution and corresponding calculation of autophagic and mitophagic events are depicted in the diagram. White arrows depict the events of autophagy.

We previously demonstrated MDF in the cells of several FA sub-groups [9]. In order to study in depth mitochondrial alterations, we performed transmission electron microscopy (TEM) following 3D reconstruction of mitochondria shape in FA vs. FA corrected cells (Figure 1A and Supplementary Figure S1A). Interestingly, we identified increased number of mitochondria with lower average volumes in FANCA and FANCC cells compared to their corrected counterparts (Figure 1A). We recapitulated these results in HEK cells converted to FA-like phenotype by siRNA knock down, wherein we observed fewer mitochondria with higher average volumes in control scRNA transfected vs. FA-like cells (Supplementary Figure S1B, S1C). These results also corroborate well with FACS analyses of average size of freshly isolated FA mitochondria (Figure 1B) and explain our previous observations of similar mitochondrial mass between FA and FA corrected cells (Supplementary Figure S2A).

One possible explanation for having these changes is the misbalance between mitochondrial fission and fusion events. This is often preceded by OS and accompanied by autophagic events [16]. Indeed, both cellular and mitochondrial ROS levels were higher in FA vs. FA corrected cells (Figure 1C). Visual inspection of TEM images with higher resolution revealed elevated levels of autophagic (mostly mitophagic) events in FA cells comparatively to corrected counterparts (Figure 1D). Thus we conclude that oxidatively stressed FA cells have higher mitochondrial fission rate with concomitant presence of mitophagic events.

### Mitophagy in FA cells is dependent on oxidative stress

**Figure 2:**
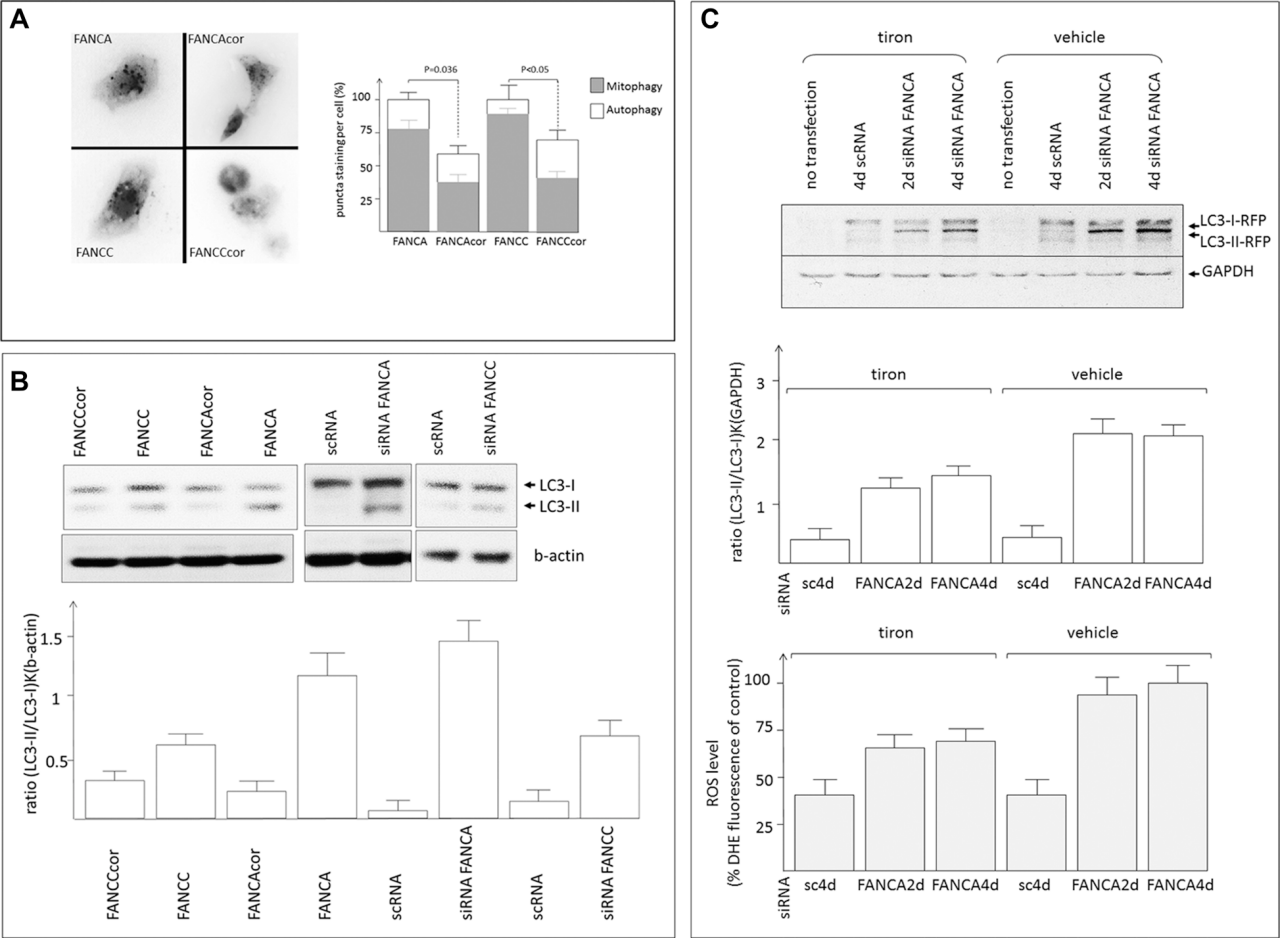
ROS-dependent mitophagy in FA cells (**A**) FA fibroblasts expressing mitochondrial GFP-TOM20 were transfected with RFP-LC3II reporter construct and the puncta structures corresponding to the events of autophagy, overlapping with GFP (Supplementary Figure S2) signals (corresponding to mitophagy) were plotted onto the diagram. (**B**) Representative immunoblot shows lipidated (LC3-II) and non-lipidated (LC3-I) forms of cell lysates from FA cells or human fibroblasts depleted of FANCA or FANCC by siRNA. Corresponding graph shows signal intensities of LC3-II/I bands of the above immunoblot. (**C**) Representative immunoblot of FANCA depleted fibroblasts with/out treatment with tiron (upper panel) and the diagram showing signal intensities of LC3-II/I bands (middle). Corresponding samples were subjected to ROS measurements and the results of three independent measurements were plotted on the diagram (bottom panel).

We also determined the number of mitochondria colocalizing with autophagy marker Atg8/LC3. Transfection of RFP-LC3 reporting construct into the above FA cells resulted in identification of a profound number of RFP-LC3 puncta structures in FA vs. FA corrected cells (Figure 2A). Consequently, co-staining of these cells with co-transfected mitochondrial GFP-TOM20 revealed significant overlap with LC3 signals, suggesting predominant occurrence of mitophagy in FA cells (Figure 2A and Supplementary Figure S2). Western blot analyses of FA cells revealed stronger signals of LC3 lipidated form (LC3-II) indicating increased autophagosomal formation (Figure 2B). In order to avoid errors linked to replication history of FA cells we depleted primary human fibroblasts for FANCA or FANCC by siRNA and demonstrated similar increase of LC3-II signals (Figure 2B). Since mitophagy is triggered by OS in a mitochondrial fission dependent manner [17], we further tested these events in the above RFP-LC3II transfected cells in the absence or presence of a ROS scavenger tiron (Figure 2C). FANCA depleted cells demonstrated a significant increase in the intensity of lipidated LC3 form comparatively to control cells and pretreatment with ROS scavenger was able to reduce LC3II/I ratio suggesting that autophagy (mitophagy) in FA-like cells is OS dependent.

### Mitophagy is impaired in FA cells

**Figure 3:**
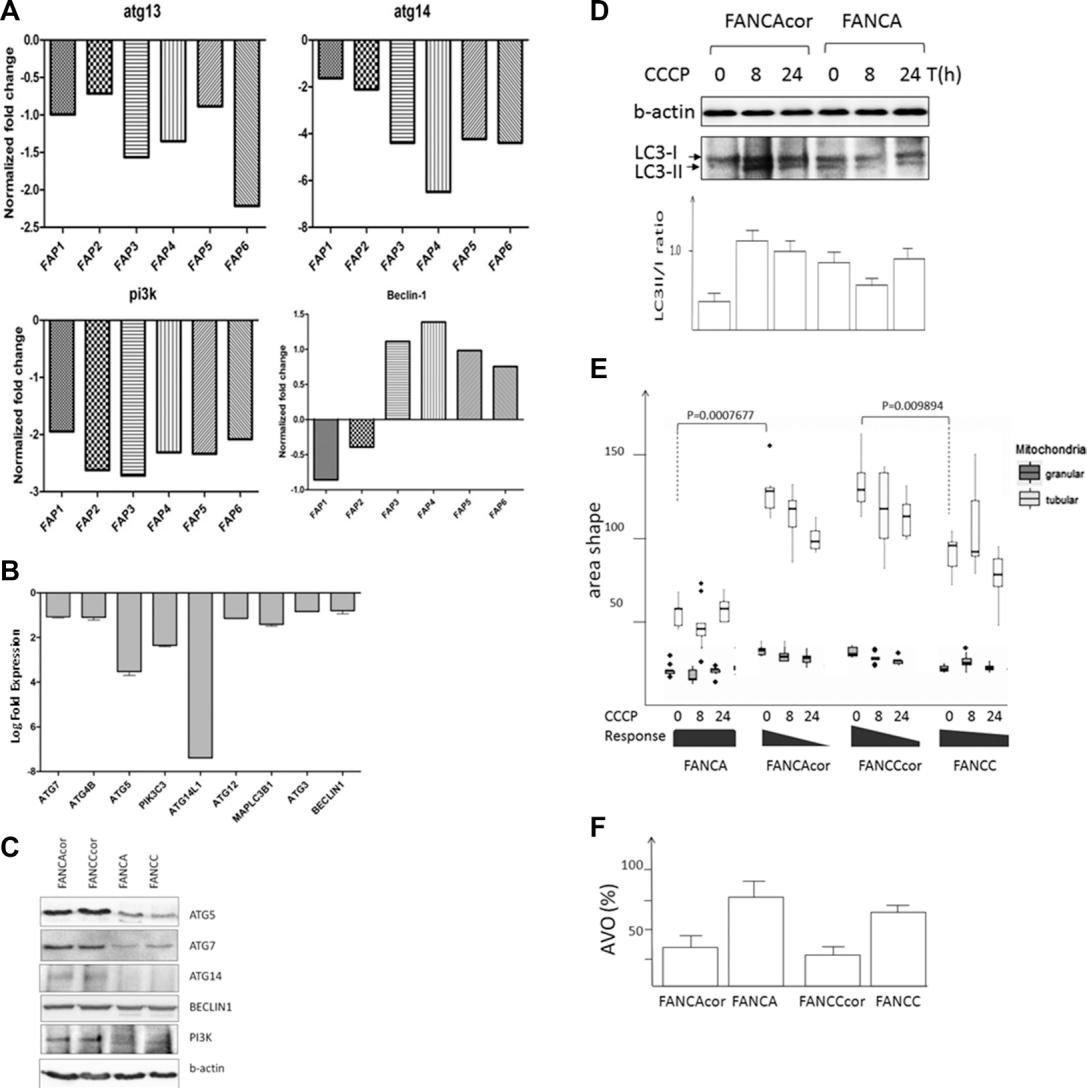
Mitophagy is impaired in FA cells (**A**) Microarray data depicting downregulation of some autophagy genes in FA patients vs. healthy individual or (**B**) in FA deficient vs. proficient cells. Total RNA isolated from peripheral blood of 6 Fanconi anemia patients from Andhra Mahila Sabha Hospital, Chennai, or from individuals with no symptoms of FA, was amplified and used for hybridization with the Human 40 K (A + B) OciChip array followed by Hybridization was performed using automated hubstation HS 4800. Hybridized chips were scanned using Affymetrix 428TM array scanner at three different PMT gains. Differentially expressed genes were filtered and the results represent the most downregulated mitochondrial genes. A threshold log fold change (LFC) of 3.0 was fixed to attain FDR of less than 0.05. The accession to the microarray datasets can be found in our earlier studies (29). (**C**) Corresponding protein profiles of FA cells. (**D**) Representative immunoblot of LC3 with FA cells treated with CCCP for the indicated time interval and diagram with signal intensities of LC3-II/I. The results are the multiple scanned (*n* = 4) of two independent blots, *P* = 0.0026 (**E**) Morphological changes of FA mitochondria upon treatment with CCCP. 2000 cells per well were stained with mitotracker-green and Hoechst and the cell images were acquired on Image Xpress Ultra scanning confocal microscope and analyzed using Cell Profiler. Statistical significance was determined by Wilcoxon rank sum test. (**F**) Percent of lysosomes and autophagolysosomes (AVO) as determined by AO staining of FA cells and corrected counterparts (*P* < 0.03, *n* = 4, *t-test*).

Our data on mitochondrial damage in FA accompanied by the induction of mitophagy could be due to increased mitochondrial biogenesis or decreased clearance of abnormal mitochondria by mitophagy, or both. Interestingly, expression of many autophagic markers, except beclin 1, were downregulated in six FA patient samples as well as in FA deficient cells (Figure 3A–3C). These data corroborate well with the qRT-PCR analysis of FANCA or FANCC cells demonstrating downregulation of some mitochondrial genes in comparison to corrected counterparts (Supplementary Figure S3). In order to explain higher levels of autophagy (mitophagy) in FA-deficient vs. FA-proficient cells and the downregulation of autophagic genes in FA-deficient cells we tested the hypotheses of impaired autophagy (mitophagy) in FA. To this end, we stimulated FA cells for autophagy by the mitochondrial uncoupler CCCP, which typically results in autophagic/mitophagic digestion of the damaged mitochondria [18]. While CCCP treatment led to increased LC3II/I ratio of FA-corrected cells, it did not increase further in FA-deficient cells (Figure 3D). Moreover, although CCCP treatment of FA corrected cells led to expected mitochondrial damage, as revealed by decreased number of tubular vs. granular mitochondria, the level of granular mitochondria after CCCP treatment did not further increase (Figure 3E and Supplementary Figure S4). These results were validated by immunofluorescent images of the above cells with RFP-LC3 reporter construct (data not shown). Formation of AVOs (lysosomes and autophagolysosomes), one of the autophagic characteristics, was quantified by flow cytometry after staining the cells with AO which accumulates in acidic spaces and fluoresces bright red. Impaired autophagy was confirmed using AO staining (Figure 3F). Altogether, these observations suggest defective mitophagy in FA-deficient cells.

### Impaired mitophagy in FA cells is dependent on mitochondrial fission

**Figure 4:**
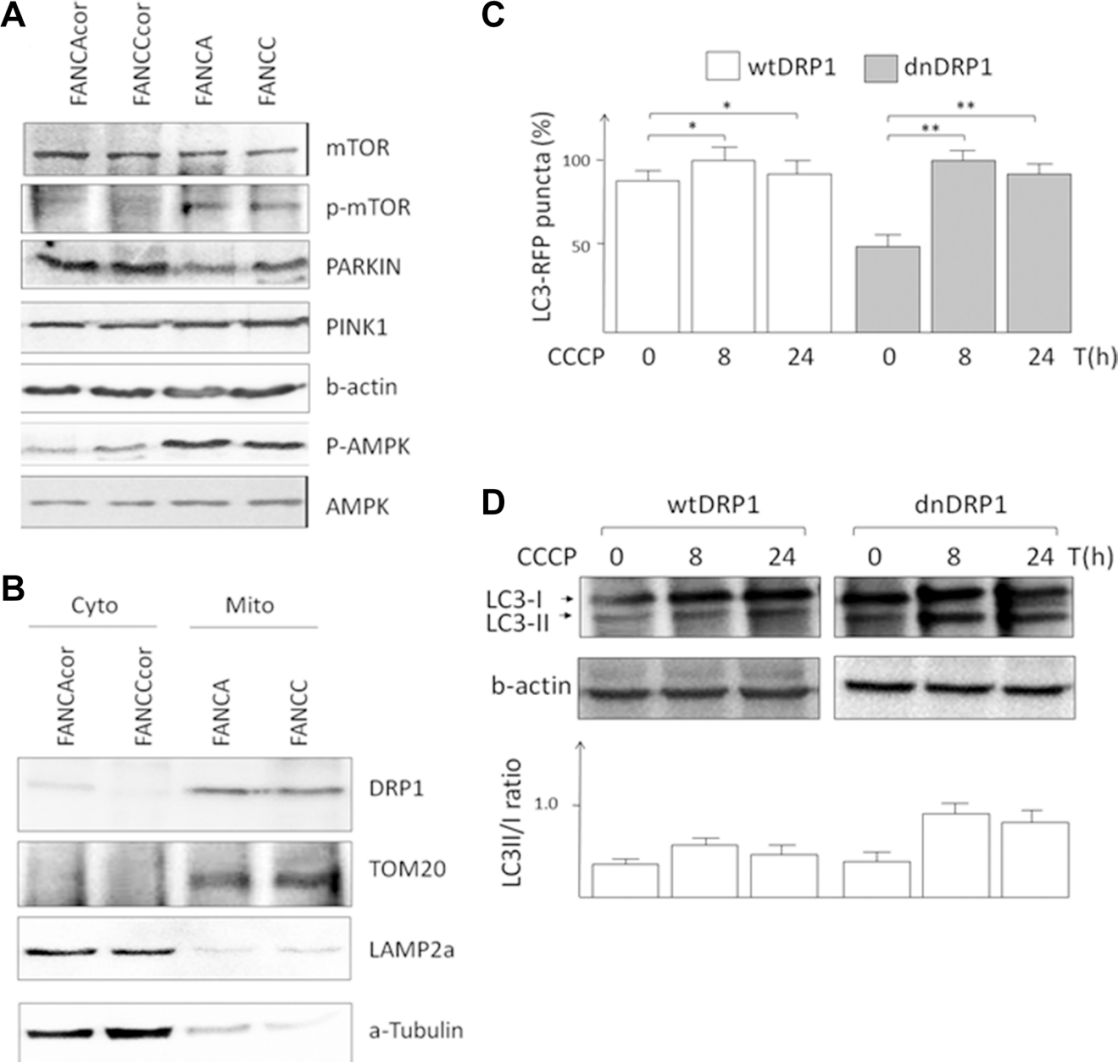
Impaired mitophagy in FA cells is dependent on mitochondrial fission (**A**) Immunoblots of possible autophagic markers. (**B**) Immunoblots of mitochondrial and cytoplasmic fractions of FA cells. (**C**) Fractions of FA cells transfected with wtDRP1 or dnDRP1 were enriched by FACS-sorting, treated with CCCP for indicated time intervals and transfected with LC3-RFP. Representative graph showing the average number of puncta signals in FANCA cells. *P < 0.05, **P < 0.03 The plasmids CMV_DRP1_IRES_mitoDsRed andCMV_DRP1dn_IRES_mitoDsRed expressing wild type and dominant negative DRP1 (DRP1dn = DRP1K38A). (**D**) Corresponding cell samples were processed for immunoblot analyses and the average signal intensities of LC3-II/I bands were plotted on a diagram.

We then assessed other factors that might potentially impair autophagy in FA. Consequently, we tested whether the defective autophagy observed in FA resulted from dysregulation of the mTOR pathway. Immunoblot analysis revealed some up-regulation of mTOR signaling in FA cells, as evidenced by the increased phosphorylation of mTOR (Figure 4A). We also tested involvement of Parkin and PINK1 however no significant change in their expression levels was observed (Figure 4A). The most interesting finding was activation of the adenosine monophosphate (AMP)–activated protein kinase (AMPK), a central metabolic sensor activated by a wide variety of mitochondrial insults. AMPK has previously been tied to mitophagy [19]. At the same time, mitochondrial fission involves the recruitment of the GTPase dynamin-related protein 1 (DRP1) from the cytosol to the mitochondrial surface to catalyze the fission reaction [20]. Here we found that DRP1 is recruited to the mitochondria of FA deficient cells (Figure 4B). Since DRP1 accumulation in mitochondria produces fission, we tested whether mitochondrial fission was required for mitophagy in FA cells. We discovered that expression of a dominant-negative variant of DRP1 (DRP1dn) but not wild type DRP1 led to reduced puncta signals of GFP-LC3 (Figure 4C) and restored CCCP-induced LC3II/I ratio (Figure 4C, 4D). Thus, we conclude that blocking mitochondrial fission may prevent the induction of mitophagy in FA cells.

## DISCUSSION

**Figure 5:**
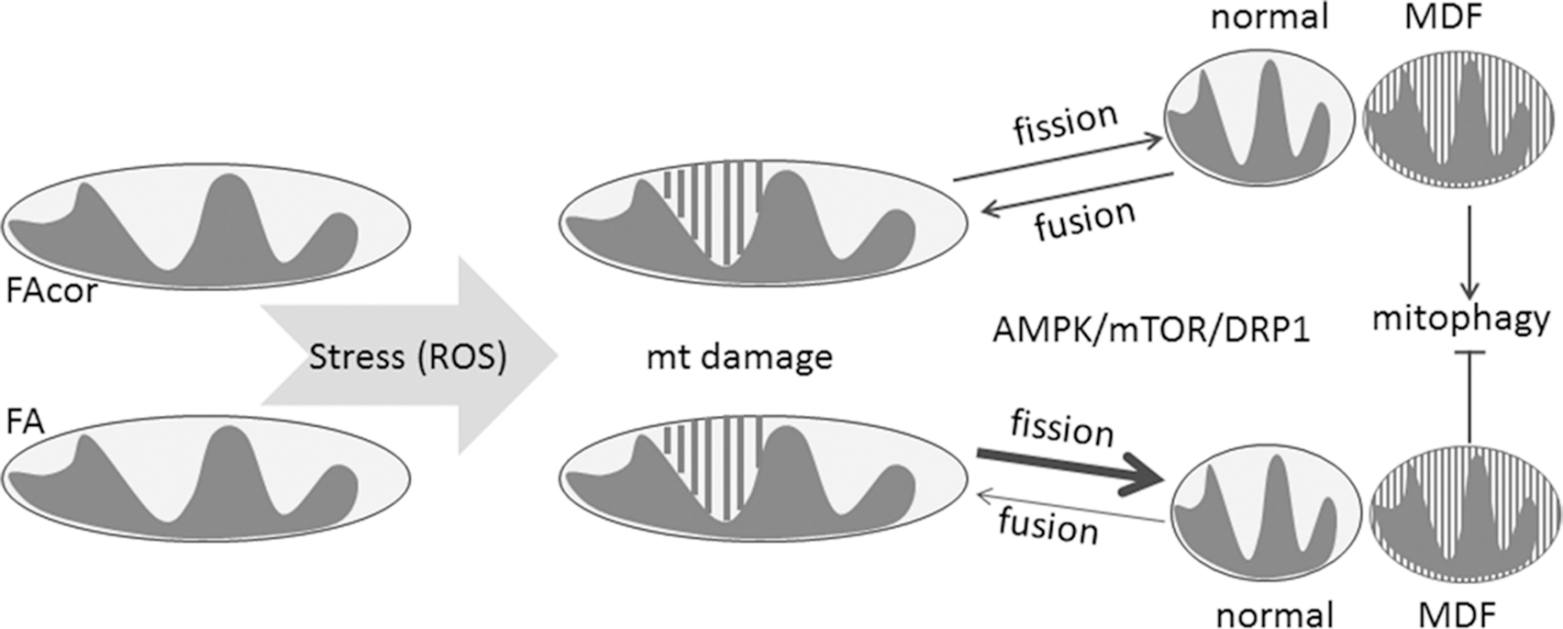
MDF in FA cells is dependent on ROS and impaired mitophagy Defects in DNA damage response FA genes result in OS which, in turn, damages mitochondrial parameters and eventually leads to MDF. In healthy cells (FAcor) dysfunctional mitochondria are removed from the intact mitochondrial reticular by mitophagy. Mitochondrial fission is necessary for the induction of mitophagy under mild OS and helps to segregate normal and damaged mitochondria. Activation of AMPK and mTOR signal cells to rapid mitochondrial fragmentation by triggering DRP1. In unhealthy condition, such as in FA, cells can not remove dysfunctional mitochondria because mitophagy is impaired. Therefore, accumulation of damaged mitochondria takes places leading to physiological consequences that impact overall FA phenotype.

Overall, the current study provides the first evidence of mitochondrial fission-dependent impaired mitophagy in FA. The working scheme depicted in Figure 5 suggests that OS, which is likely occurred due to defects in DNA damage response FA genes, may create MDF. Removal of dysfunctional mitochondria from the intact mitochondrial network is thought to prevent further damage [11] and mitochondrial fission is necessary for the induction of mitophagy under mild OS [16]. Activation of AMPK and mTOR is required for cells to undergo rapid mitochondrial fragmentation perhaps, through a newly identified mitochondrial fission factor which serves as a mitochondrial outer-membrane receptor for DRP1 [21]. Moreover, DRP-1-dependent apoptosis can trigger mitochondrial fission [22]. However. FA cells are not apoptotic but rather pre-apoptotic [23] hence the role of mitochondrial fission is likely dependent on other pathways.

Regardless of the possible mechanisms, there are several questions that need to be addressed. The first one is what is the initial trigger for MDF and accompanied autophagy in FA. Does OS occur as a result of defective FA genes and initiates MDF with all corresponding consequences or there are some intrinsic factors making FA mitochondrial damage? To answer that part, a cybrid technology can be applied [24]. In particular, fusion of enucleated FA cells with mitochondria-depleted FA corrected cells, and vice versa, would determine the impact of defective FA nuclear genome to MDF. Some studies on Ataxia Telangiectasia Mutated (ATM) disease suggest that these events are not necessarily coupled as MDF-mediated ATM activation may take place without evidence of DNA damage [25]. The second important question is how common the above phenomena of MDF and impaired autophagy might be for the DDR-related disorders. Recently, we have reviewed that many syndromes with the affected DNA damage and repair machinery share common clinical phenotypes with mitochondria-related disorders [26]. It remains to be seen if impaired autophagy will be also found in some other DDR disorders as, for example, A-T-like disorder or Bloom syndrome.

The third important question is whether MDF and impaired mitophagy may, at least in part, explain cancer occurrence in FA patients who are at a greater risk of developing acute myeloid leukemia (AML) and head and neck squamous cell carcinoma. As autophagy/mitophagy is thought to be dysregulated in some cancers, one can speculate that defective mitophagy in FA may be linked to cancer predisposition. It is well possible that mitochondria of lymphoblastoid cells experiencing hyperoxya are primarily affected by high ROS microenvironment and inability to remove damaged mitochondria result in cancer occurrence. In turn, cells in solid tumors are subjected to prolonged hypoxia. To this end, defective mitophagy that serves as an adaptive metabolic response, which is necessary to prevent increased levels of ROS and cell death, may in principle play a role in solid tumors formation. For instance, in some CRC models under hypoxic conditions, impaired clearance of protein oligomers (and resulting apoptosis) have been observed, whereas culturing cells under normoxic conditions protected cell viability and proliferation. Our and others studies previously showed that FA cells were rather pre-apoptotic [23]. Therefore, an additional hit would be required for FA cells to become apoptotic. This could be increased ROS level or infection disease. Finally, upon submission of the current paper, we also became aware of a study showing involvement of FANCA protein in mitophagy and immunity [27]. The study also confirms that DDR defective genes are not solely responsible for FA phenotype and therefore adds some additional insight on how mitophagy in FA can be altered. If so, targeting mitophagy can become a possible strategy for the anticancer therapy in FA.

## MATERIALS AND METHODS

### Antibodies and chemicals

The full list of antibodies and chemicals is provided in Supplementary Table S1A. For all the mitochondrial assays, chemicals used were of the highest purity and did not contain divalent ions, especially Ca^2+^ and Zn^2+^. Double-distilled water treated with catalase was used for mitochondrial experiments.

### Cell cultures, and preparation of cell extracts

The full description of cell lines and culturing conditions is provided in Supplementary Table S1B. Briefly, two types of human FA deficient and corrected cells were received in December 2015: lymphoblastoid cells (abbreviated as EUFA, obtained and recently tested in Dr.Surralles' lab) and fibroblasts (abbreviated by the specific FA gene, obtained and recently tested in Dr. Smogorzewska's lab). For mitochondrial studies, cells were grown without antibiotics.

### Plasmids, transient transfection and lentiviral transduction

Transient transfections were performed using FuGENE HD (Roche Applied Science) according to the manufacturer's instructions. The plasmids pEGFP-C1-rLC3 or pRFP-LC3 were purchased from AddGene. The plasmids CMV_DRP1_IRES_mitoDsRed andCMV_DRP1dn_IRES_mitoDsRed (gift from Dr. Reichert AS) expressing wild type and dominant negative DRP1 (DRP1dn = DRP1K38A) were used to produce lentiviruses in HEK293T cells using pLp1, pLp2, pLp3 packaging system (Invitrogen) according to the published protocol (http://www.ericcampeau.com/manuals.html).

### Microarray studies and real time PCR analyses

The accession to the microarray datasets can be found in our earlier studies [30]. RNA from FANCA, FANCC and corresponding corrected counterparts were utilized. In parallel, RNA samples from FA patients were compared with control healthy individuals. Validation was carried out using qRT-PCR essentially as described earlier [28].

### siRNA experiments

siRNA depletion experiments were performed as described earlier [9]. For each of the FA genes, a library of FA siRNAs have been screened and siRNA showing more than 70% knock-downeffect was selected for further experiments.

### Fluorescence microscopy to study mitochondrial morphology

2000 cells per well were stained with mitotracker-green and Hoechst. For autophagy experiments cells were acquired on a Nikon 1 AR inverted Microscope using a 40× objective and 561 nm (50 mW) excitation wavelength. For morphological changes, cell images were taken with Image Xpress Ultra (Molecular Devices) automated scanning confocal microscope with Plan/APO 40×/0.95 objective.

### Image acquisition and statistical analyses

For image analyses two independent channels were obtained – nuclei stained with Hoechst as DAPI channel; and mitochondria stained with mitotracker green as FITC channel. 9 independent sites per each well in 96 well plate were acquired. Images were analyzed using Cell Profiler open source software (www.cellprofiler.org). First nuclei were detected by Otsu segmentation in DAPI channel. Cells were detected in FITC channel smoothed with Gaussian filter as secondary objects arising from primary objects – nuclei. Detected mitochondria in FITC channel were related to each parental object – cell. Mitochondria were scored based on their shape factor eccentricity, describing the circular – ellipsoid shape of object. As a threshold we used factor 0.8. Mitochondria having factor higher than 0.8 are considered as vesicular, mitochondria with lower factor are considered as vesicular. For each cell was calculated total area of tubular/granular mitochondria.

Before statistical analysis images were filtered to eliminate over/under segmented images, based on number of detected nuclei per image. Images with more than 80 nuclei were removed. For statistical comparison of two mitochondria populations Wilcoxon rank sum test was performed.

### Transmission electron microscopy (TEM)

TEM was performed essentially as described previously [9]. For siRNA experiments, cells were co-transfected with GFP pcDNA3.1 vector and processed for FACS sorting. Enriched (96–98%) fraction of cells has been used for EM imaging.

### Isolation and purification of mitochondria

Mitochondrial fractionation has been performed essentially as described earlier [9]. Approximately 100 million cells grown in antibiotic free medium were used for each experiment. Mitochondrial pellets were immediately aliquoted and kept frozen at −80°C.

### Western blot analysis

The procedure was performed essentially as described earlier [9].

### Measurement of ΔΨm

Measurement and quantification of ΔΨm has been performed essentially as described earlier [9]. Briefly, 25,000 cells per well in quadruplicates were plated onto 96-well plate and 1 μm of JC-1 (Molecular Probes) was added to each well. Fluorescence wavelengths, adjusted to blank controls, were measured using Tecan Infinite F500 (Tecan Systems, Germany).

### Measurements of intracellular and mitochondrial ROS

Measurements of intracellular ROS were performed with fluorogenic dye H2DCF-DA (Molecular Probes, Eugene, OR, U.S.A.) as earlier described [9]. Mitochondrial ROS was detected with MitoSOX red reagent according to the manufacturer's protocol (Invitrogen) following procedure described previously [9].

### Acridine orange (AO) assay

100,000 cells plated in 6-well plate were stained with 1 μg/ml AO orange for 15 min. Cells were washed once with PBS, detached from the plate processed for the FACS analyses. Total fluorescence of AO was normalized to DMSO-treated cells to show the AO fold changes.

### Measurement of ATP production

ATP production was measured using Promega kit (Promega, Madison, WI, USA) as described in the manufacturer's manual. Samples were prepared in quadruplicates and experiments were repeated 3 times.

## SUPPLEMENTARY MATERIALS FIGURES AND TABLE





## Abbreviations

OS: oxidative stress
ΔΨm: mitochondrial membrane potential
MDF: mitochondrial dysfunction
ROS: reactive oxygen species
RedOX: KD knock-down
CCCP: RFP red fluorescent protein
GFP: green fluorescent protein
DRP1: Dynamin-1-like protein
LC3: light chain 3
AO: acridine orange
CCCP: carbonyl cyanide m-chlorophenyl hydrazone
