# Supplementary material for: Impaired mitophagy in Fanconi anemia is dependent on mitochondrial fission
Source: Oncotarget. 2016 Aug 9;7(36):58065–74. doi: 10.18632/oncotarget.11161 (PMC5295412; doi:10.18632/oncotarget.11161)
Supplement: Supplementary file 1 [file oncotarget-07-58065-s001.pdf]

Impaired mitophagy in Fanconi anemia is dependent on mitochondrial fission

Supplementary Materials

REFERENCES

1. Lyakhovich A, Surrallés J. Constitutive activation of caspase-3 and Poly ADP ribose polymerase cleavage in fanconi anemia cells. Mol Cancer Res. 2010; 8:46–56. doi: 10.1158/1541-7786.MCR-09-0373.
2. Bogliolo M, Lyakhovich A, Callén E, Castellà M, Cappelli E, Ramírez MJ, Creus A, Marcos R, Kalb R, Neveling K, Schindler D, Surrallés J. Histone H2AX and Fanconi anemia FANCD2 function in the same pathway to maintain chromosome stability. EMBO J. 2007; 26:1340–51.
3. Rickman KA, Lach FP, Abhyankar A, Donovan FX, Sanborn EM, Kennedy JA, Sougnez C, Gabriel SB, Elemento O, Chandrasekharappa SC, Schindler D, Auerbach AD, Smogorzewska A. Deficiency of UBE2T, the E2 Ubiquitin Ligase Necessary for FANCD2 and FANCI Ubiquitination, Causes FA-T Subtype of Fanconi Anemia. Cell Rep. 2015; 12:35–41. doi: 10.1016/j.celrep.2015.06.014.
4. Yonghwan Kim, Gabriella S. Spitz, Uma Veturi, Francis P. Lach, Arleen D. Auerbach, Agata Smogorzewska Regulation of multiple DNA repair pathways by the Fanconi anemia protein SLX4. Blood. 2013; 121:54–63.
5. Stephanie Huelga C, Anthony Vu Q, Justin Arnold D, Tiffany Liang Y, Patrick Liu P, Bernice Yan Y, John Paul Donohue, Lily Shiue, Shawn Hoon, Sydney Brenner, Manuel Ares Jr, Gene W. Yeo Integrative genome-wide analysis reveals cooperative regulation of alternative splicing by hnRNP proteins Cell Rep. Cell Rep. 2012; 1: 167–178.

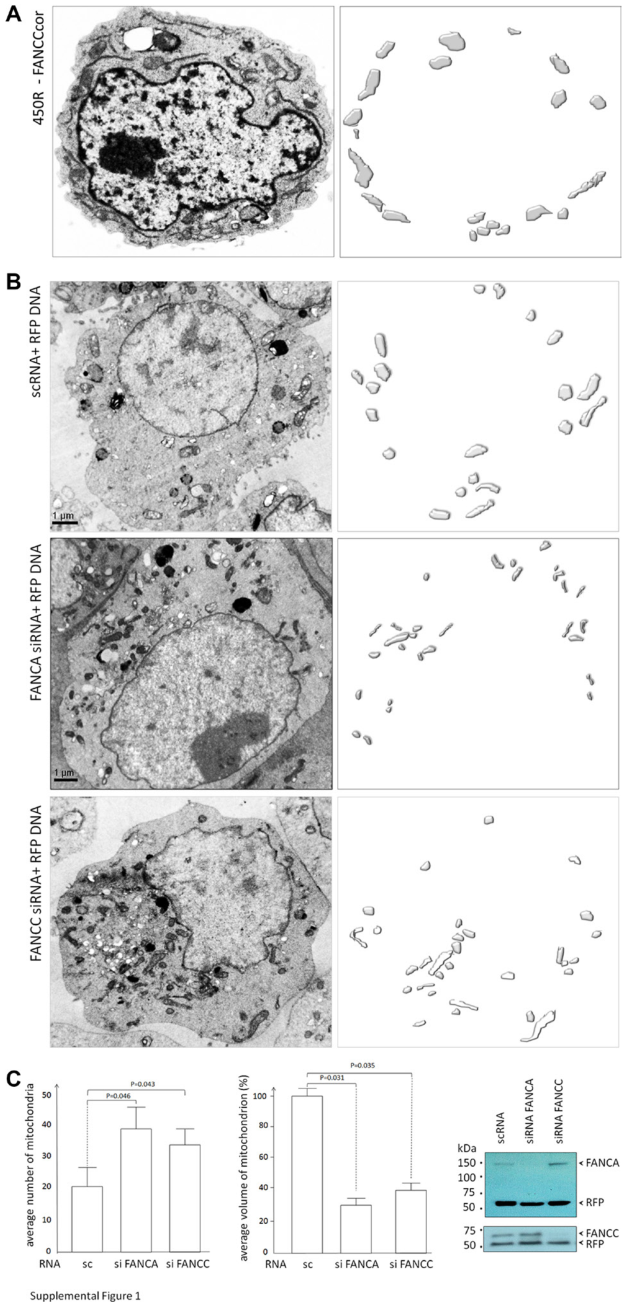

Supplementary Figure S1: Mitochondria in FA and FA corrected cells differ in volumes and numbers. (**A**) Representative image of FANCC or (**B**) FA-like cells obtained from siRNA depletion of HEK cells. siRNA depletion experiments were performed as described earlier (9). For each of the FA genes, a library of FA siRNAs have been screened and siRNA showing more than 70% knock-down effect was selected for further experiments. TEM was performed essentially as described previously (9). Cells were transfected with corresponding siRNA or scRNA with 1:1 ratio of RFP pDNA following FACS-sorting to enrich transfected fractions (**C**) TEM images from ~9 serial sections 0.2 µm thick were stacked, aligned, and 3D reconstructed. Eight random sections were taken into analysis for each cell lines. Data represent average ± SEM. (**D**) The efficiency of siRNA knock-down was confirmed by immunoblotting Western with corresponding antibodies.

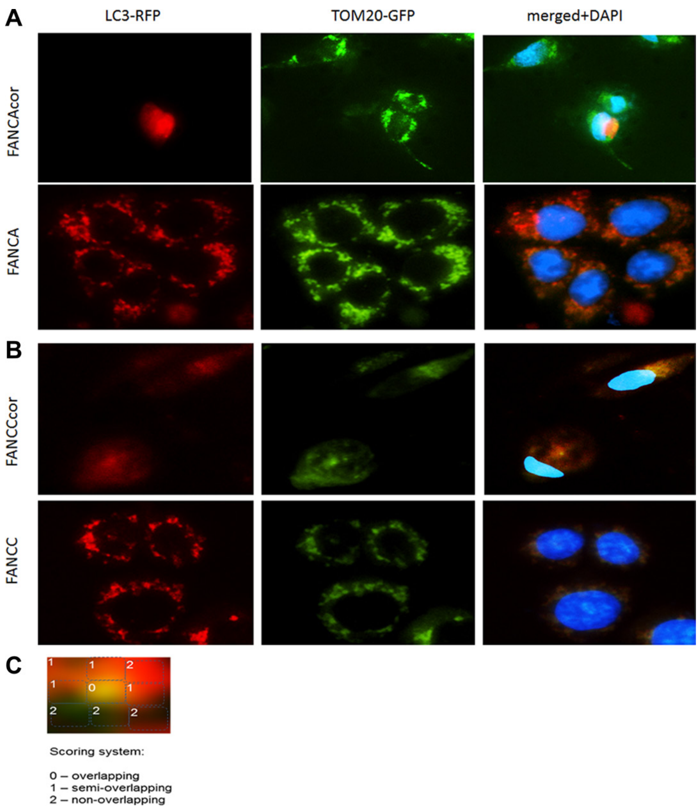

Supplementary Figure S2: Similar mitochondrial mass and the events of autophagy (mitophagy) in FA cells. (**A**) FA fibroblasts and corrected counterparts from subgroups A and C were pre-labeled with mito-tracker green and after isolation of mitochondria from the same number of cells, the signal intensities have been measured to define the mitochondrial mass difference. FANCA, FANCAcor (**B**), FANCC, FANCCcor (**C**) cells were transfected with GFP-TOM20 and RFP-LC3II reporting construct 2 days before the experiments. After adding DAPI, living cells were processed for imaging (NIKON, ×40) and the puncta structures (correspond to the events of autophagy), as well as overlapping with TOM20 green signals (correspond to the events of mitophagy), were plotted onto the diagram. (**D**) The scoring system was based on 75–100% overlapping (score 0), 25–75% semi-overlapping (score 1) and 0–25% non-overlapping (score 2).

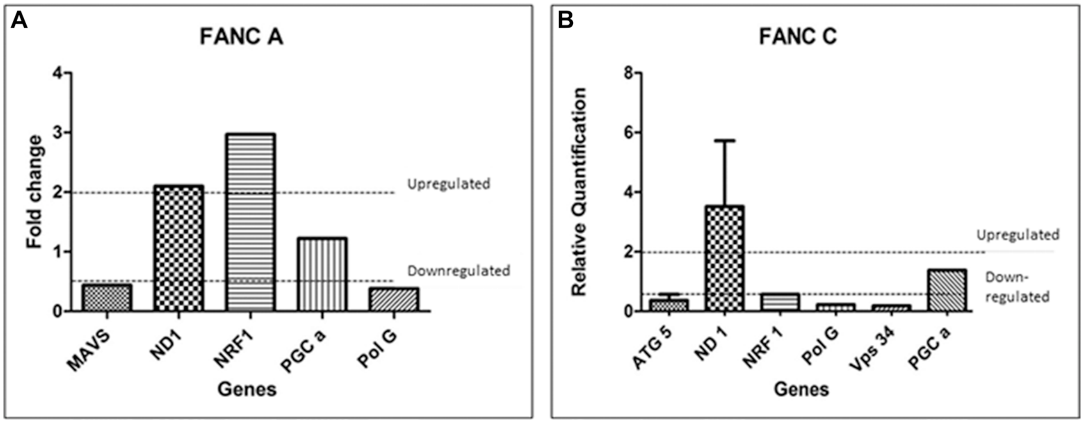

Supplementary Figure S3: Gene expression levels of mitochondrial genes in FA cells. The qRT-PCR data showing downregulation of ATG and some mitochondrial genes in FA deficient vs. proficient cells.

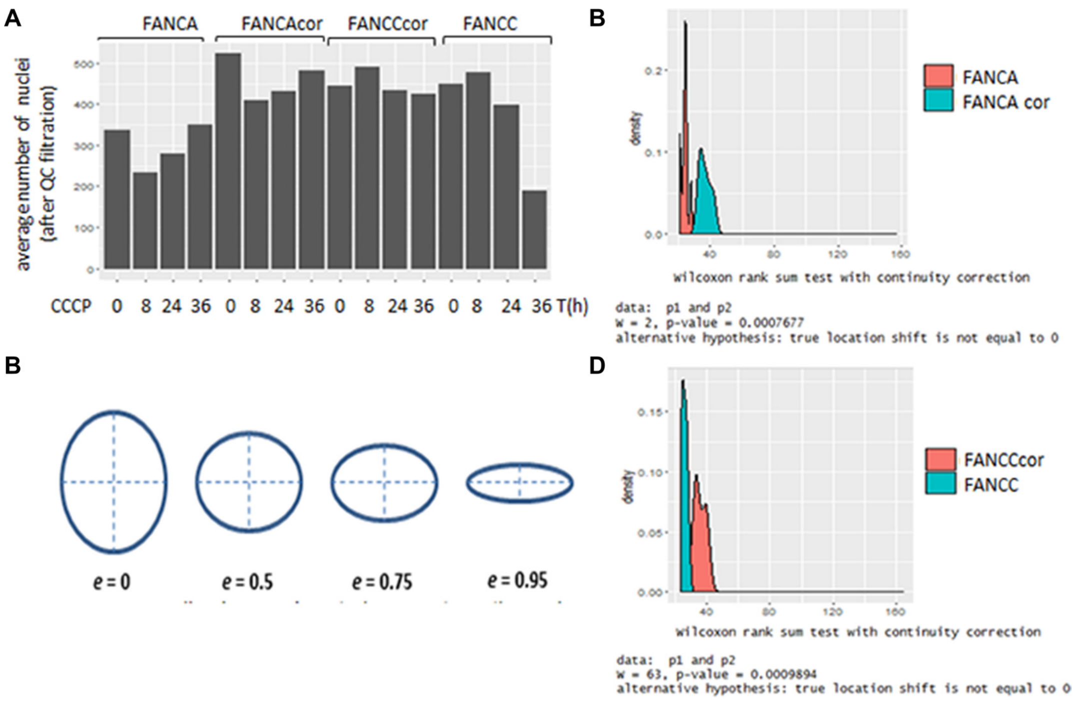

Supplementary Figure S4: Morphological changes of mitochondria in FA cells. (**A**) FA and FA corrected cells stained with Hoechst and mitotracker green. Images acquired on Image Xpress Ultra (Molecular Devices) automated scanning confocal microscope with Plan/APO 40×/0.95 objective. Two independent channels were obtained – nuclei stained with Hoechst as DAPI channel; and mitochondria stained with mitotracker green as FITC channel. We acquired 9 independent sites per each well in 96 well plate. Images were analyzed using Cell Profiler open source software (www.cellprofiler.org). First nuclei were detected by Otsu segmentation in DAPI channel. Cells were detected in FITC channel smoothed with Gaussian filter as secondary objects arising from primary objects – nuclei. Detected mitochondria in FITC channel were related to each parental object – cell. (**B**) Mitochondria were scored based on their shape factor eccentricity, describing the circular – ellipsoid shape of object. As a threshold we used factor 0.8. Mitochondria having factor higher than 0.8 are considered as vesicular, mitochondria with lower factor are considered as vesicular. For each cell was calculated total area of tubular/granular mitochondria. (**D**) After separation mitochondria into two categories, the morphology was measured and the data were grouped by image and presented by boxplot. Wilcoxon rank sum test with continuity correction was applied to measure *P*-values

Supplementary Table S1: List of antibodies, chemicals (A) and cells (B) with specific conditions used in the current study. See Supplementary_Table_S1
